# Radiographic imaging in relation to the mandibular third molar: a survey among oral surgeons in Sweden

**DOI:** 10.1007/s00784-021-04189-9

**Published:** 2021-10-01

**Authors:** Josefine Cederhag, Anna Truedsson, Per Alstergren, Xie-Qi Shi, Kristina Hellén-Halme

**Affiliations:** 1 Department of Oral and Maxillofacial Radiology, Faculty of Odontology, Malmö University, SE-205 06 Malmö, Sweden; 2 Department of Oral & Maxillofacial Surgery and Oral Medicine, Faculty of Odontology, Malmö University, SE-205 06 Malmö, Sweden; 3 Department of Orofacial Pain and Jaw Function, Faculty of Odontology, Malmö University, SE-205 06 Malmö, Sweden; 4 Specialized Pain Rehabilitation, Skåne University Hospital, Lund, Sweden; 5 Section of Oral and Maxillofacial Radiology, Department of Clinical Dentistry, University of Bergen, Postboks 7804, 5020 Bergen, Norway

**Keywords:** Clinical decision-making, Dental radiography, Oral surgical procedures, Third molar, Tooth extraction

## Abstract

**Objectives:**

To query the experience of oral surgeons concerning referral routines and preferences for radiographic imaging modality before surgical removal of mandibular third molars and investigate factors that influence imaging modality preferences.

**Materials and methods:**

Members of the Swedish Association of Oral and Maxillofacial Surgeons (*n* = 280) were invited to participate in a web-based digital survey concerning their experiences and use of three imaging modalities in pre-surgical assessment of mandibular third molar removal. The survey comprised multiple-choice questions and four cases depicted in images; respondents reported whether they would supplement the cases with other images and, if so, from which modality.

**Results:**

The response rate was 64%. Panoramic radiographs were most commonly used in pre-surgical planning (response options: *always* or *often*), significant difference between professions (*p* = 0.039), and considered to facilitate treatment planning (87%), as was CBCT (82%); for 51%, CBCT reduced post-operative complications. Preferred modality for localizing the mandibular canal was fairly evenly distributed and for non-complex case, significant difference between subgroups of OMFS surgeons was found (*p* = 0.003) as to preference for intraoral radiographs.

**Conclusions:**

A majority of respondents received a report within 2 weeks of their referral for CBCT and would read the report and view the images before surgery. Image modality preference differed depending on case complexity, with a greater perceived need for CBCT. Profession and practical experience affected choice.

**Clinical relevance:**

Choice of imaging modality in mandibular third molar assessment is also important from dose delivery and social economy standpoints.

**Supplementary Information:**

The online version contains supplementary material available at 10.1007/s00784-021-04189-9.

## Introduction

Removal of mandibular third molars is a common surgical procedure in dentistry. Before removal of the tooth, a clinical examination and, often, a radiographic investigation are done to evaluate the position and anatomy of the third molar and its relation to surrounding vital structures. This information aids the clinician in decision-making on therapy choices with confidence and reduces the risk of surgical complications. Surgical removals of mandibular third molars are also often associated with post-operatively side effects such as oedema, pain, trismus, and oral dysfunction. Alveolar osteitis, infections, and damage to the adjacent tooth are examples of complications. A serious complication is sensory disturbance caused by injury to the inferior alveolar nerve (IAN) [1].

Three radiographic imaging modalities are commonly used in pre-surgical assessment: intraoral radiography, panoramic radiography, and cone beam computed tomography (CBCT), each with their own advantages and drawbacks. Intraoral radiographs provide high contrast and spatial resolution and can thus depict fine anatomical structures and pathological changes. However, overlapping anatomical structures in the bucco-lingual dimension can make it difficult to assess tooth anatomy and the location of the mandibular canal using the parallax technique. Furthermore, placement of radiographic receptors posteriorly in the third molar area can be challenging.

A panoramic radiograph has lower spatial resolution and is thus less detailed than intraoral radiographs; however, it provides a useable overview of the teeth, bone, and anatomical structures in the maxillofacial area and is considered a useful method for many third molar cases before surgical removal [2].

CBCT is a three-dimensional modality with the unique ability to precisely define the spatial relationship between the roots and the IAN. CBCT examinations, however, are more expensive due to the relatively high cost of the technique and resources used [3, 4], and it delivers a higher radiation dose to the patient compared to two-dimensional radiography [5]. European evidence-based guidelines on the use of CBCT in dentistry include justification and optimization strategies [5]. These guidelines [5], and the well-known principle of as low as reasonably achievable (ALARA) [6], indicate that use of CBCT, as part of pre-operative assessment of mandibular third molars, should be restricted to select cases where two-dimensional radiography is unable to accurately depict the relationship, if there is a close inter-relationship between IAN and the third molar and when a decision of removal has been made.

A CBCT examination to supplement panoramic imaging or intraoral radiographs may be justified if one or more radiographic signs of close relationship are present or if two-dimensional radiography does not allow assessment of the position of IAN [7–9]. The accuracy of panoramic radiographs for evaluating third molar root morphology and number of roots is limited [10], and several studies have considered CBCT images to be more accurate [11, 12]. Moreover, Hauge et al. [13] reported that CBCT more accurately identified direct contact to the mandibular canal than panoramic radiographs. Specific signs detected on panoramic radiographs can suggest a close inter-relationship and should be considered for further treatment choice [14–17]. During recent years, the Swedish Radiation Safety Authority [18] has observed an increase in registered CBCT units. The same phenomenon has been reported from Switzerland, yet panoramic imaging was still regarded as the reference investigation in dentistry. Nevertheless, it was found that wisdom teeth were the most common indication for CBCT [19]. Another study in the USA [20] investigated the use of CBCT in imaging of impacted teeth among other indications in oral and maxillofacial surgery and found an overall trend of using CBCT instead of panoramic imaging. To reflect the expert opinion, that study proposed guidelines and the use of CBCT in the case of impacted teeth was considered to be “usually indicated” [19]. Accordingly, clinicians presumably use CBCT more frequently today, probably due to the higher confidence expected in decision-making, supported by a more exact assessment of three-dimensional anatomical relationships. However, use of CBCT has been shown to neither substantially change the treatment plan [8, 21] nor reduce the presence of permanent sensory disturbances [8, 22, 23] or other post-operative complications [24] compared with use of panoramic imaging for pre-surgical assessment. Thus, CBCT should not be routinely used [5, 8, 9, 22].

Koon et al. (2006) surveyed the use of radiographic methods among oral and maxillofacial surgeons in Australia to determine the relationship of IAN and the third molar and reported that panoramic radiographs were most commonly used, even though many considered panoramic radiographs to have limitations in precisely determining the interrelation [25]. In contrast, Matzen et al. (2016) observed that the most common examination in Danish general dental clinics before surgical removal of the third molar was intraoral radiographs alone, even though 36% were insufficient [26].

In 2012, the European Commission published guidelines [5] on the use of CBCT. To our knowledge, no study has investigated the approach and judgments of clinicians concerning radiographic imaging before surgical removal of mandibular third molars using patient cases in a questionnaire survey. Thus, this study aimed to investigate referral routines and preferences in radiographic examination before surgical removal of the mandibular third molar based on the experience of dental professionals and to investigate what factors influence the choice of radiographic modality for general dentists, residents, and specialists in oral and maxillofacial surgery.

## Materials and methods

Swedish regulations required no ethical approval of this study.

All members of the Swedish Association of Oral and Maxillofacial Surgeons with a listed email address (*n* = 280) were invited by email to participate in a web-based questionnaire. Eligible members included licensed specialists in oral and maxillofacial surgery (OMFS surgeons), residents in oral and maxillofacial surgery (OMFS residents), and general dental practitioners (GDPs) working in the oral and maxillofacial surgical field. To be included, participants needed to be clinically active. The invitation informed participants about the study and about the voluntary and anonymous nature of participation. No questions concerned sensitive personal information. Each participant received a personal web link guiding them to the questionnaire. The survey was published in May 2020 and closed in September 2020. Three reminders were mailed to eligible participants. We used Sunet Survey Software (Artologik Survey&Report Version 4.3, Artisan) to build the survey. To enhance the quality of the questions and reduce measurement errors, three dentists in the OMFS field pre-tested the survey and suggested structural and linguistic revisions to simplify the wording of the questions.

### Questionnaire

The survey comprised two parts with a total of 22 multiple choice questions:

I. Eight questions concerned respondent characteristics, including gender; age; profession (OMFS surgeons/OMFS residents/GDPs); type of clinical practice such as private, public, hospital, and university; experience in oral surgery; and experience in surgical removal of mandibular third molars.
II. Fourteen questions dealt with radiological aspects of the respondents’ practice, such as equipment availability and referral routine. The respondents were asked to estimate their perceived use of three x-ray modalities (intraoral, panoramic, and CBCT radiography) and their experiences with these in pre-surgical assessment based on four aspects: facilitating the treatment planning for removal, reducing post-operative complications related to anatomy and position, avoiding removals, and changing treatment strategy.

**Fig. 1:**
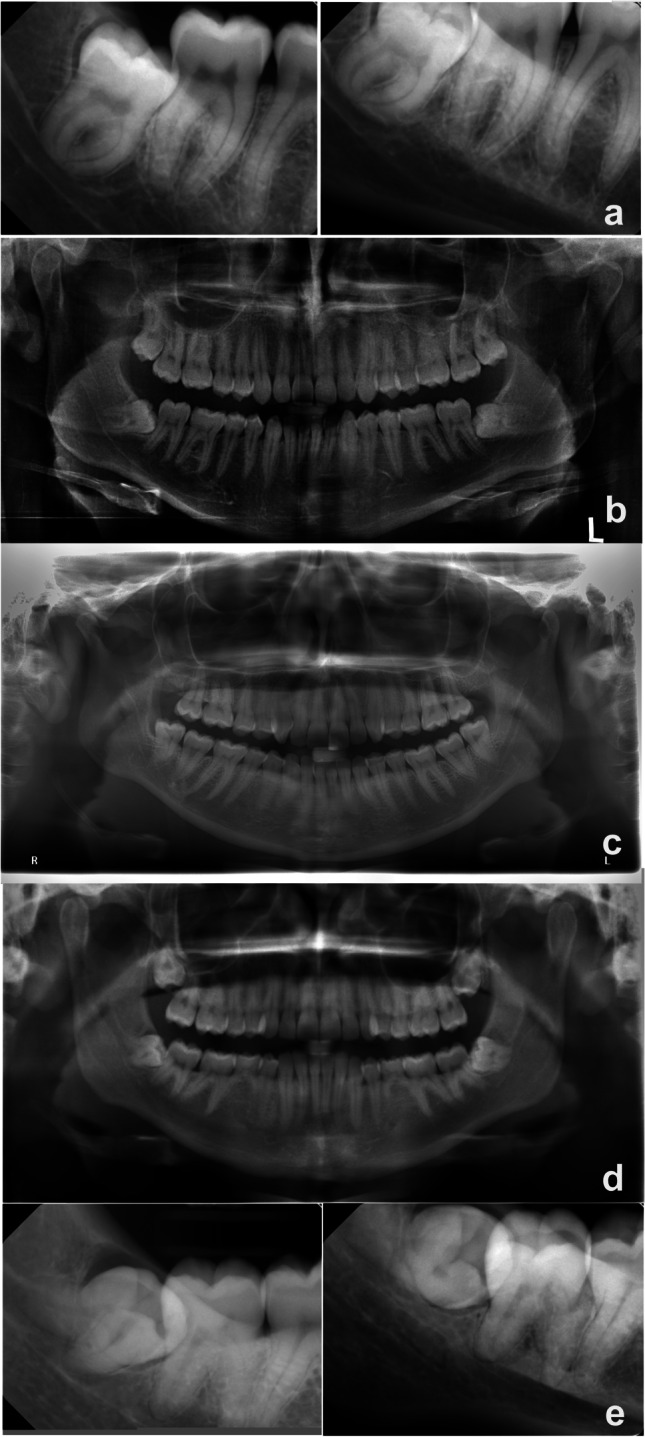
The radiographs presented in the survey. **a** Case I. **b** Case II. **c** Case III. **d** Panoramic radiograph for case IV. **e** Intraoral radiographs for case IV

The questionnaire also included four cases described solely with radiographs (Fig. 1). Case selection was performed by a committee consisting of a junior OMF radiologist, senior OMF surgeons, and senior OMF radiologists to ensure the best clinical relevance. All four cases had third mandibular molar roots overlapped with IANs, assessed based on conventional radiographs. They were considered commonly seen cases in clinical practice and represent degree of difficulties in the justification process of CBCT. Case 1 represented a relatively easy justification process in which the root anatomy and localization of IAN could be assessed by two intraoral radiographs using parallax technique. Cases 2 and 3 were similar; both had panorama and the preference of desired radiographic examination was expected to vary in these two cases. Case 4 represented a complex inter-relationship between roots and IAN since the superior border of the mandibular canal was not visible in the panoramic radiograph; thus, the need of CBCT was expected to be apparent.

The images were anonymous and written informed consent was received from each patient. Each case comprised a panoramic radiograph and/or intraoral radiographs of the mandibular third molar with questions concerning the radiographic information: whether, in the judgment of the respondent, the images gave adequate information for decision-making before removal or if further imaging was needed, and if so, which kind (intraoral, panoramic, or CBCT). Three aspects were to be considered: position of the mandibular canal, third molar root anatomy, and relation to the adjacent second molar.

### Statistical analysis

Data were imported into Microsoft Excel 2016 (Microsoft Corporation, Redmond, WA, USA), and frequency analysis was used to sort, count, and compare respondent characteristics for the different questions. The OMFS surgeons were divided into two subgroups: OMFS-1 (surgeons licensed before year 2000) and OMFS-2 (licensed after 2000) since in Sweden, the dental CBCT was introduced in the early 2000s. Cross-tabulations, the Pearson chi-squared test, and Fisher’s exact test were used to compare responses within different professions and the Statistical Package for the Social Science (IBM SPSS, version 23 for Windows) to test for significant differences. Significance was set at *p* ≤ 0.05.

## Results

The response rate was 64% (*n* = 179). Of those, 23 were not clinically active and thus excluded from the survey; 10 did not answer all the questions. The remaining respondents comprised OMFS surgeons (103, 71%), OMFS residents (23, 16%), and GDPs (20, 14%). Among the OMFS surgeons, 76% (*n* = 78) had been licensed in 2000 or later (range for all OMFSs: before 1980 to after 2016). Seventy-five percent (*n* = 110) of the respondents were male and 64% (*n* = 93) were aged 45 years or older (range: 25 to > 65 years). Sixty-nine percent (*n* = 99) worked more than 30 h per week (range for the entire study cohort: < 10 to ˃ 30 h/week) with the oral and maxillofacial surgery, and almost equally common, the respondents surgically removed fewer than five third molars per week (53, 37%) on average or between five and ten third molars per week (51, 35%; range for the study cohort: < 5 to ˃ 30 removals/week). In 45% (*n* = 65), the respondents had more than one practice type and, most commonly, practiced at hospitals (91, 63%).

### Radiology referral for mandibular third molar imaging

Availability of panoramic and CBCT units at the clinic was 85% (*n* = 122) and 70% (*n* = 100), respectively. Regardless of examination, two-thirds responded that they received the radiology report within 2 weeks (14, 67% and 95, 66%). For CBCT referrals, 93% (*n* = 133) of the respondents always asked for a report and 92% (*n* = 128) both read the report and viewed the images; 6% (*n* = 8) only viewed the images, and 2% (*n* = 3) only read the report. Thirty percent (*n* = 42) stated that they usually did not or never waited for the radiology report before surgical removal of the tooth.

### Intraoral, panoramic, and CBCT radiographs for pre-surgical assessment

**Fig. 2:**
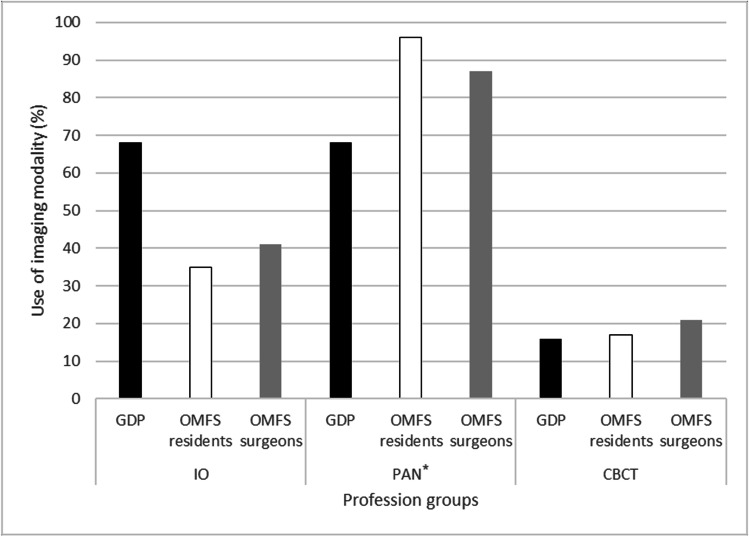
Self-reported use (response option: *always* or *often*) of radiographic imaging modalities before surgical removal of mandibular third molars. The study cohort comprised three groups: general dental practitioners (GDPs), oral and maxillofacial residents (OMFS residents), and surgeons (OMFS surgeons). *Significant difference between profession groups according to panoramic imaging (*p* = 0.039) (Fisher-Freeman-Halton exact test, exact sig 2-sided). IO, intraoral radiographs; PAN, panoramic radiographs; CBCT, cone beam computed tomography

The modality that respondents would most likely use (response option: *always* or *often*) before planned surgical removal of a third molar was panoramic radiographs (123, 86%), followed by intraoral radiographs (62, 43%) and CBCT (28, 20%). Figure 2 shows preferences by profession in radiographic imaging modality. OMFS-1 preferred intraoral radiographs (13, 54%) compared with OMFS-2 (28, 36%), as did those who surgically removed fewer than five mandibular third molars per week (27, 51%). Between-group differences, however, are not significant.

### Pre-surgical decision-making and post-operative complication reduction

Table 1 presents the respondent’s experience concerning which imaging modality facilitates the treatment planning for third molar removal and reduces post-operative complications related to anatomy and position (response option: *always* or *often*) by profession*.* Most respondents experienced that panoramic radiographs (123, 87%) and CBCT (116, 82%) facilitated the treatment planning for removal compared with intraoral radiographs (60, 42%). More—but not significantly more—in the OMFS-1 subgroup (12, 52%, response option: *always* or *often*) had found intraoral radiographs to be also helpful in the treatment planning for removal compared with the OMFS-2 subgroup (29, 38%). The majority of the respondents (72, 51%, response option: *always* or *often*) experienced that information obtained from CBCTs helped reduce post-operative complications related to anatomy and position, while panoramic radiographs fulfilled this purpose best for 37% (*n* = 52) and intraoral radiographs for 16% (*n* = 23). No modality provided information that had more than a minor impact (response option: *always* or *often*) on changing treatment strategy or reversing the decision to remove the third molar. For intraoral radiographs, only 2% (*n* = 3) of the respondents felt the imaging information made a large impact on treatment strategy and 3% (*n* = 5) and that the information reversed the decision to remove the third molar; the corresponding numbers for panoramic radiographs were 4% and 4% (*n* = 6), and for CBCT, 10% (*n* = 15) and 6% (*n* = 9).

**Table 1:** Experiences of whether radiographic imaging modalities (response option: *always* or *often* chosen) facilitated the treatment planning of third molar extraction and reduced post-operative complications related to anatomy and position. General dental practitioners (GDPs), oral and maxillofacial residents (OMFS residents), and surgeons (OMFS surgeons)

Profession	Preferred radiographic imaging modality					
	Facilitates treatment planning for removal			Reduces post-operative complications		
	IO (%)	PAN (%)	CBCT (%)	IO (%)	PAN (%)	CBCT (%)
GDP	57.9	78.9	84.2	31.6	36.8	63.2
OMFS residents	34.8	100.0	91.3	8.7	30.4	34.8
OMFS surgeons	41.0	85.0*	79.0	15.2	38.0	52.0

^*^Significant difference between OMFS-1 (100.0%) and OMFS-2 (80.5%); *p* = 0.019 (Fisher’s exact test)

*IO*, intraoral radiographs; *PAN*, panoramic radiographs; *CBCT*, cone beam computed tomography

### Radiographic imaging cases in the survey

**Fig. 3:**
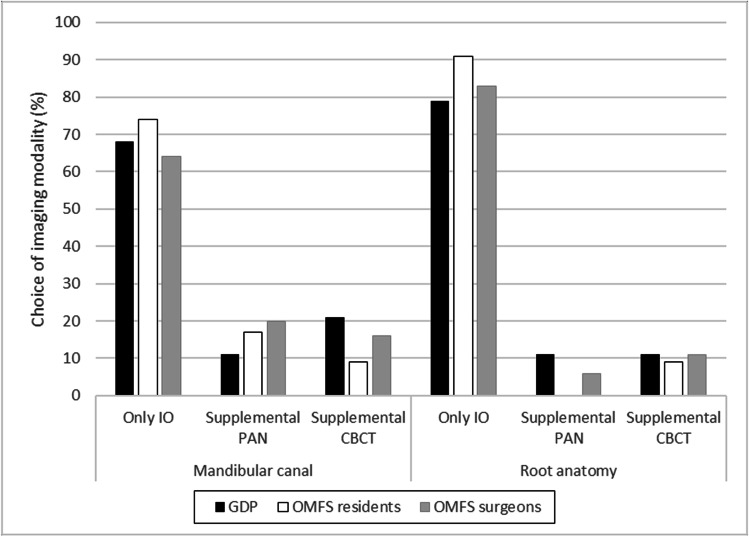
Case 1. Judgments of whether the intraoral radiographs from the radiographic examination sufficed or if other imaging modalities were needed to assess the position of the mandibular canal and the root anatomy of the third molar before extraction. The study cohort comprised three groups: general dental practitioners (GDPs), oral and maxillofacial residents (OMFS residents), and surgeons (OMFS surgeons). PAN, panoramic radiographs; CBCT, cone beam computed tomography

Table 2 presents the radiographic imaging modality best suited—in the eyes of the respondents—for judging the position of the mandibular canal, the anatomy of the third molar root, and the relation of the third molar root to the adjacent tooth before surgical removal for each of the four cases. In case I, a majority in each professional group judged the intraoral radiographs to be sufficient in assessing both the position of the mandibular canal and root anatomy (Fig. 3). However, as to the position of the mandibular canal, significantly fewer in the OMFS-1 (12, 52%) than the OMFS-2 (52, 68%) subgroup judged so (*p* = 0.003). Further, a higher proportion of specialists in the OMFS-1 subgroup felt intraoral images to be insufficient for judging the position of the mandibular canal (9, 39%) and the root anatomy (5, 22%) and would have liked supplemental images with CBCT compared to in the OMFS-2 subgroup (7, 9% and 7, 9%). These differences between the subgroups were not significant.

**Table 2:** In the eyes of the respondents, the radiographic imaging modality or modalities best suited for judging the position of the mandibular canal, the anatomy of the third molar root, and the relation of the third molar root to the adjacent tooth before surgical removal. For cases 1 − 3, images from only one modality were presented; for case 4, images from 2 modalities were presented

Survey case and radiographic images	Response choice	Mandibular canal (%)	Root anatomy (%)	Relation to adjacent tooth (%)
Case 1 Intraoral	Yes, IO suffices	66.2	83.1	68.3
	No, supplement with PAN	18.3	5.6	17.6
	No, supplement with CBCT	15.5	11.3	14.1
Case 2 Panoramic	Yes, PAN suffices	22.5	63.4	91.7
	No, supplement with IO	33.8	19.0	4.9
	No, supplement with CBCT	43.7	17.6	3.5
Case 3 Panoramic	Yes, PAN suffices	28.2	61.3	93.0
	No, supplement with IO	37.3	19.0	3.5
	No, supplement with CBCT	34.5	19.7	3.5
Case 4 Panoramic and intraoral	Yes, PAN and IO suffice	23.9	21.1	68.3
	No, supplement with CBCT	76.1	78.9	31.7

*IO*, intraoral radiographs; *PAN*, panoramic radiographs; *CBCT*, cone beam computed tomography

**Fig. 4:**
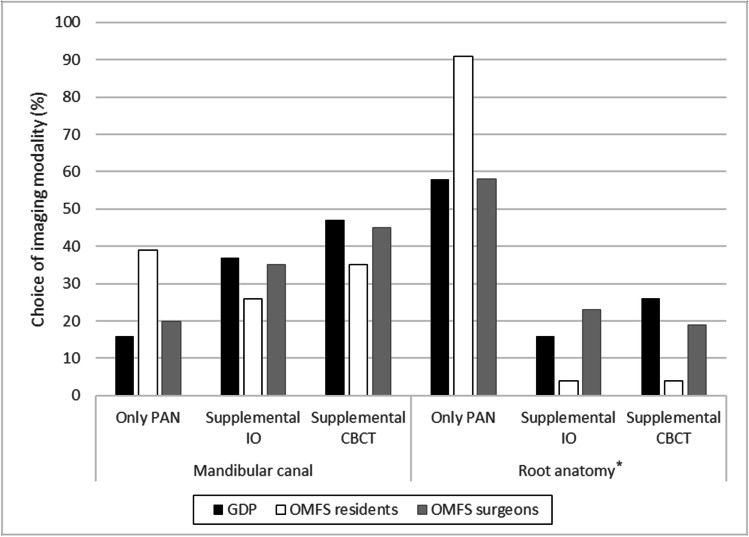
Case 2. Judgments of whether the panoramic radiographs from the radiographic examination sufficed or if other imaging modalities were needed to assess the position of the mandibular canal and the root anatomy of the third molar. The study cohort comprised three groups: general dental practitioners (GDPs), oral and maxillofacial residents (OMFS residents), and surgeons (OMFS surgeons). *Significant difference between profession groups (*p* = 0.032) (Fisher-Freeman-Halston exact test, exact sig 2-sided). IO, intraoral radiographs; CBCT, cone beam computed tomography

**Fig. 5:**
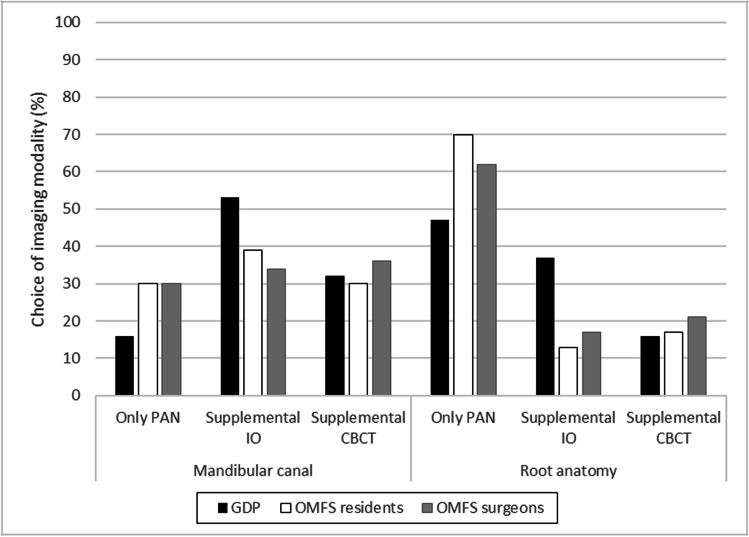
Case 3. Judgments of whether the panoramic radiographs from the radiographic examination sufficed or if other imaging modalities were needed to assess the position of the mandibular canal and the root anatomy of the third molar. The study cohort comprised three groups: general dental practitioners (GDPs), oral and maxillofacial residents (OMFS residents), and surgeons (OMFS surgeons). IO, intraoral radiographs; CBCT, cone beam computed tomography

In cases II (Fig. 4) and III (Fig. 5), opinions were more evenly distributed as many judged panoramic radiographs were insufficient for making judgments on position of the mandibular canal without supplemental images; however, most experienced them as sufficient for assessing the anatomy. The OMFS-1 subgroup would have chosen to supplement the panoramic radiographs with CBCT for judging root anatomy (case II: 6, 26%; case III: 8, 35%), higher than in the OMFS-2 subgroup (both case II and case III: 13, 17%). These differences between the subgroups, however, were not significant.

For case IV, a majority in each professional group agreed that the radiographic information was insufficient for assessing the position of the mandibular canal, the root anatomy, and their relation (Table 2). Therefore, further examination with CBCT was considered needed, especially among the OMFS residents (19, 83%) regarding the mandibular canal and among the OMFS residents (20, 87%) and GDPs (18, 95%) regarding the root anatomy.

## Discussion

The present study found that most of the respondents considered panoramic radiographs as a pre-surgical examination before removal of a mandibular third molar to be helpful and experienced that panoramic radiographs and CBCT facilitated treatment planning for third molar removal. Respondents also believed that the additional information from CBCT imaging reduced post-operative complications related to anatomy and position better than intraoral and panoramic radiographs. The majority of respondents considered CBCT justified in more complicated cases, such as case 4. Interestingly, radiographic imaging seemed to have little impact on treatment strategy, regardless of modality. The preferred modality for other surgical aspects differed according to profession and practical experience.

Questionnaires are a well-documented, often-used research tool [27–30]. Online surveys have become popular due to participant-friendly aspects such as convenience and ease compared with their paper-based counterparts. However, the limitations of questionnaire studies include the reliability and generalizability of results due to low participation rates, which are common, and to sampling, coverage, and measurement errors [31]. The response rate in the present study was in line with reports from similar studies [25, 32, 33], but a complete dropout analysis could not be done due to the anonymous distribution of the questionnaire. Still, the respondents were all members of the National Association of Oral and Maxillofacial Surgeons in Sweden. Also, according to statistics from the Swedish National Board of Health and Welfare, the gender and age distribution of the OMFS surgeons’ group were representative for the entire group of licensed OMFS surgeons in Sweden [34].

The present study explored the preferred choice of dental professionals with surgical expertise (GDPs, OMFS residents, and surgeons) for radiological assessment. Patient perspective was not considered in the current study design. One aim of the present study was to establish the process of decision-making and to what extent knowledge of the efficacy of the imaging modalities was involved. Further, in a future study, it would be most interesting to investigate the underlying factors that affect respondents’ decisions, such as clinical experience or knowledge of radiation protection in terms of justification.

Most (92%) would read the radiology report and view the radiographs. This is considerably higher than what Hol et al. [33] reported, although their study was based on CBCT requests for several diagnostic tasks. It is, however, tempting to speculate why one-third in the present study began patient treatment before receiving the CBCT report from the radiologist. Hol et al. [33] reported a similar phenomenon in Norway, where specialist clinics were more likely to begin treatment before receiving the radiology report than general dental clinics. Strindberg et al. [35] found in their questionnaire survey of general and specialist clinics where considerably more general clinics participated (46% in Sweden vs 17% in Norway) that almost all of the responding Swedish dentists (96%, compared with 59% in Norway) awaited the radiology report before beginning any treatment. Long waiting times may partly explain why a third of the dentists did not wait for the report.

The use of radiographic modalities, especially intraoral and panoramic radiographs, seemed to differ by profession and, to some extent, practical experience. Most (86%) of the respondents (response options: *always* or *often*) would use panoramic radiographs in pre-surgical assessment of the mandibular third molars if possible. This is expected since the panoramic unit is commonly accessible, the image provides a useable overview, and in many cases, it is sufficient for a pre-surgical examination of the third molar [2]. Also, it is a cost-effective choice [4]. A survey among OMFS surgeons in Australia (*n* = 105; 72 responded) found that almost all respondents (97%) frequently used a panoramic radiograph for determining the relationship of IAN and third molar in cases of probable third molar removal, while considerably fewer used intraoral radiographs or CBCT imaging [25]. In the present study, use of panoramic radiographs differed significantly between professions, with the OMFS surgeons and residents using panoramic radiographs more frequently than GDPs. In this context, general dental clinics have been found to prefer intraoral radiographs before third molar removal [26]. This is similar to the findings of the present study, where both GDPs and OMFS-1 used intraoral radiographs more frequently than professionals in the other groups. An explanation for this could be that GPDs and specialists in the OMFS-1 subgroup are more familiar with intraoral radiographs and intraoral x-ray units are available at any clinic.

Panoramic and CBCT images were preferable in facilitating treatment planning for third molar removal compared with intraoral radiographs. This was especially true for panoramic radiographs among OMFS residents and in the OMFS-1 subgroup, and the difference between the OMFS-1 and OMFS-2 subgroups was significant. Again, a larger part of GDPs and the OMFS-1 subgroup, in contrast to younger OMFS surgeons and residents, found that intraoral radiographs facilitated treatment planning than the other two modalities. Further, the finding that intraoral radiographs facilitated treatment planning less well than panoramic radiographs is an interesting finding since intraoral images depict structures sharply and distinctly with a higher resolution and diagnostic accuracy than panoramic images. By applying the parallax technique, localization of IAN is possible using two intraoral images, which is not possible with most panoramic machines. The preference for panoramic radiographs over intraoral radiographs was probably due to the overview of the posterior part of the mandible and the easiness of image capturing. The panoramic radiograph presents an overview, and the extraoral exam is perceived as convenient for both patient and clinician.

Koon et al. [25] observed in their survey of Australian OMFS surgeons that, compared with panoramic or intraoral radiographs, CBCT images were widely considered to provide more accurate and sufficient information for determining the interrelation of the IAN and the third molar; respondents were familiar with, and users of, all three modalities. Due to its ability to image the bucco-lingual section, CBCT images have also proven, compared with panoramic radiographs, to be superior for evaluating the number of roots and their morphology [11, 12]. This information, of higher accuracy and amount, most likely improves clinician confidence in treatment planning. Thus, we expected that the respondents would feel that CBCT investigations, as part of the pre-surgical assessment, would be likely to reduce post-operative complications better than the other two modalities. It appears reasonable to believe that knowledge of the three-dimensional anatomy (including morphology, number of roots, and proximity between the roots and IAN) is valuable, but still, the clinical situation for each patient is a prominent factor.

In contrast, Guerrero et al. [11] reported that post-operative complications did not decrease when CBCT was used compared to panoramic imaging. Moreover, the European Academy of DentoMaxilloFacial Radiology (EADMFR) [8] recently published a position paper based on a literature review and concluded that there is good evidence that CBCT fails to reduce sensory disturbances of IAN and other post-operative complications that require revisits. The position paper also concluded that in most cases, patient outcome was the same, regardless of whether panoramic or CBCT images had been obtained [8]. Additionally, Matzen et al. [21] showed that CBCT seldom seems to affect the treatment plan or the outcome of the third molar removal to any large degree, which was also in line with the present study. A study by Mendonça et al. [36], however, concluded that although diagnostic changes due to CBCT imaging could lead to modifications of the treatment plan, the decision to remove the tooth would be unaffected. Furthermore, a pilot study [37] concluded that CBCT imaging improved risk assessment and, hence, led to significant changes in surgical approach compared with panoramic radiographs. In this particular study [37], the authors did not report about the treatment follow-up, however; thus, the final outcome is unknown.

The preferred imaging modality during pre-surgical assessment differed according to the profession and practical experience of the respondent. OMFS residents seemed to generally have good experience of and a high opinion of panoramic radiographs while GPDs tend to prefer intraoral radiographs when more information is needed. In case I, the majority of the GDPs and OMFS residents were satisfied with the intraoral radiographs, as was a significantly larger share of the OMFS-2 subgroup than of the OMFS-1 subgroup. We also observed that in most cases considering root anatomy, a larger share of the OMFS-1 subgroup seemed to prefer CBCT compared with the other groups. Hence, it seems reasonable to assume that, over the years, they have encountered more complicated cases, such as unexpected root anatomy during extraction, and experienced the need for more information.

Moreover, the study showed that the respondents had different opinions depending on the complexity of the case, especially concerning the position of mandibular canal. The results also indicated that the more complicated the case, the higher the need for CBCT. This was most clearly seen in case 4.

Finally, due to the growing use of CBCT imaging [18–20], it is critical that its advantages and disadvantages are carefully evaluated, especially in the areas of radiation doses to patients and economic costs. Notably, the cost of CBCT imaging for assessment of impacted third molars is approximately four times higher [4], and the radiation dose, many times higher, than that for a panoramic radiograph [38, 39]. Petersen et al. [4] concluded that CBCT seldom reduces the direct (e.g., equipment, staff, and overhead) or indirect (e.g., patient) costs of surgery with its attendant pre- and post-surgical treatment. Consequently, the three-dimensional advantage of CBCT comes at a cost. Reduction in unnecessary radiation exposure to patients and expense to society are critical factors to consider before referring a case for radiographic imaging of the mandibular third molar. It must be kept in mind that the common goal in radiology, and dentistry in general, is to reduce radiation exposure to the lowest threshold that still delivers reliable radiographic information, in line with the ALARA principle [6]. CBCT may be indicated if the IAN and the roots are in close proximity and two-dimensional imaging alone is unable to provide accurate information on the position of the IAN [5, 7, 8], or if it is believed that CBCT imaging could change the treatment plan or treatment outcome [9]. The European Commission states in guidelines that a routine use of CBCT is not advisable [5]. According to regulations in Sweden, OMFS are not allowed to answer for any CBCT investigations in their practice. The choice of radiographic modalities and image interpretation shall be evaluated and performed by an oral and maxillofacial radiologist [18] according to the request from referral dentists.

From a radiology viewpoint, individual indications must be considered. Competitive alternatives to CBCT imaging that often suffice include panoramic radiographs, intraoral radiographs, or a combination of these [7, 9].

## Conclusion

The majority of OMFS residents and surgeons and of GDPs practicing oral surgery in Sweden followed the prescribed referral routine, but one-third did not, for CBCT examination before mandibular third molar removal, by reviewing the report from the radiologist before starting patient treatment. Preference of radiographic imaging modality varied depending on sub-specialization and previous professional experience. A higher tendency to prefer CBCT over intraoral and panoramic imaging was observed in more complex cases of the mandibular third molar.

## Supplementary Information

Below is the link to the electronic supplementary material.

Supplementary file1 (PDF 4839 KB)
