# Supplementary material for: Radiographic imaging in relation to the mandibular third molar: a survey among oral surgeons in Sweden
Source: Clin Oral Investig. 2021 Oct 1;26(2):2073–83. doi: 10.1007/s00784-021-04189-9 (PMC8816342; doi:10.1007/s00784-021-04189-9)
Supplement: Supplementary file 1 — Supplementary file1 (PDF 4839 KB) [file 784_2021_4189_MOESM1_ESM.pdf]

Q1

Respondent and occupation

Q2

Your gender

- Male
- Female
- Other

Q3

Your age

- 24-34 years
- 35-44 years
- 45-54 years
- 55-64 years
- 65+ years

Q4

When did you graduate from dental school?

- Before 1980
- 1980-1989
- 1990-1999
- 2000-2009
- 2010-2015
- After 2015

Q5

When did you become a licensed oral and maxillofacial surgeon?

- ○ Before 1980
- ○ 1980-1989
- ○ 1990-1999
- ○ 2000-2009
- ○ 2010-2015
- ○ After 2015
- ○ Are not a licensed oral and maxillofacial surgeon
- ○ Are a resident in oral and maxillofacial surgery

Q6

How many hours per week do you work with oral and maxillofacial surgery (OMFS)?

- ○ Less than 10 hours/week
- ○ 10-20 hours/week
- ○ 20-30 hours/week
- ○ More than 30 hours/week
- ○ Are not clinically active

Q7

In what type of practice do you work?

Multiple choices may apply

- ☐ Private specialist practice
- ☐ Private general practice
- ☐ Public specialist practice
- ☐ Public general practice
- ☐ OMFS practice at hospital
- ☐ OMFS practice at university
- ☐ Orofacial Medicine
- ☐ If other, specify

____

Q8

How many extractions/surgical removals of mandibular third molars per week do you estimate to perform in average?

Quote the number during an average week (ignore any contingents due to Covid -19)

- Less than 5
- 5-10
- 10-20
- 20-30
- More than 30

Q9

Have you completed continued education in panoramic imaging?

(Notice; This is a requirement in Sweden for dentists who capture and interpret paroramic imaging independently)

- Yes
- No

-- PAGE BREAK --

Q10

Access to panoramic unit

Access to panoramic unit
If this question **is exactly**
- No

THEN PERFORM THE FOLLOWING ACTION

Show the following questions:
- To where do you send the referral for panoramic examination?
- How long does it take for you to receive a radiology report concerning an investigation of a mandibular third molar?

Q11

Do you have a panoramic x-ray unit at your practice?

If more than one practice type, please select the one where you perform most extractions/surgical removals of mandibular third molars

- Yes
- No

Access to panoramic unit
If Do you have a panoramic x-ray unit at your practice? **is exactly**
- No

THEN PERFORM THE FOLLOWING ACTION

Show this question

Q12

To where do you send the referral for panoramic examination?

- Private practice
- Public practice
- In hospital
- At university

Access to panoramic unit
If Do you have a panoramic x-ray unit at your practice? **is exactly**
- No

THEN PERFORM THE FOLLOWING ACTION

Show this question

Q13

How long does it take for you to receive a radiology report concerning an investigation of a mandibular third molar?

- Less than 2 weeks
- 2-4 weeks
- More than 4 weeks
- Don't receive any report, evaluate the radiograph myself

-- PAGE BREAK --

Q14

Access to CBCT

Access to CBCT
If this question **is exactly**
- No

THEN PERFORM THE FOLLOWING ACTION

Show the following questions:
- To where do you send the referral for CBCT examination?

Q15

Do you have a CBCT unit at your practice?

If more than one practice type, please select the one where you perform most extractions/surgical removals of mandibular third molars

- Yes
- No

Access to CBCT

If Do you have a CBCT unit at your practice? **is exactly**

- No

THEN PERFORM THE FOLLOWING ACTION

Show this question

Q16

To where do you send the referral for CBCT examination?

- Private practice
- Public practice
- In hospital
- At university

Q17

How long does it take for you to receive a radiology report concerning an investigation of a mandibular third molar?

- Less than 2 weeks
- 2-4 weeks
- More than 4 weeks
- Don’t receive any report, evaluate the images myself

-- PAGE BREAK --

Q18

Radiology report concerning the mandibular third molar

Q19

If a supplemental radiological investigation is needed do you usually specify what kind of investigation you are requesting?

- Yes
- No

Radiology report of CBCT
If this question **is either**
- Yes, always
- Yes, when I ask for it

THEN PERFORM THE FOLLOWING ACTION

Show the following questions:
- When receiving the CBCT report from the radiologist, what do you usually do?
- Do you begin the extraction/surgical removal of the mandibular third molar before receivning the report from the radiologist?

Q20

Do you receive a radiology report of CBCT?

- ○ Yes, always
- ○ Yes, when I ask for it
- ○ No

Radiology report of CBCT
If Do you receive a radiology report of CBCT? **is either**
- Yes, always
- Yes, when I ask for it

THEN PERFORM THE FOLLOWING ACTION

Show this question

Q21

When receiving a CBCT report from the radiologist, what do you usually do?

- ○ Read the report
- ○ Read the report and evaluate the images
- ○ Don't read the report, evaluate the images myself

Radiology report of CBCT
If Do you receive a radiology report of CBCT? **is either**
- Yes, always
- Yes, when I ask for it

THEN PERFORM THE FOLLOWING ACTION

Show this question

Q22

Do you begin the extraction/surgical removal of the mandibular third molar before receivning the report from the radiologist?

- ○ Never
- ○ Usually not
- ○ Usually
- ○ Always

-- PAGE BREAK --

Q23

Radiographic modalities

Q24

Estimate how often you use each modality before surgical removals of the mandibular third molar

	Always	Often	Sometimes	Seldom	Never
Intaoral radiographs	○	○	○	○	○
Panoramic radiographs	○	○	○	○	○
CBCT	○	○	○	○	○

Q25

Do you experience that intraoral radiographs as pre-surgical assessment

	Always	Often	Sometimes	Seldom	Never	Don't want to answer
facilitating the treatment planning for removal	○	○	○	○	○	○
avoiding removals	○	○	○	○	○	○
changing treatment strategy	○	○	○	○	○	○
reducing post-operative complications related to anatomy and position	○	○	○	○	○	○

Q26

Do you experience that panoramic radiographs as pre-surgical assessment

	Always	Often	Sometimes	Seldom	Never	Don't want to answer
facilitating the treatment planning for removal	○	○	○	○	○	○
avoiding removals	○	○	○	○	○	○
changing treatment strategy	○	○	○	○	○	○
reducing post-operative complications related to anatomy and position	○	○	○	○	○	○

Q27

Do you experience that CBCT as pre-surgical assessment

	Always	Often	Sometimes	Seldom	Never	Don't want to answer
facilitating the treatment planning for removal	◯	◯	◯	◯	◯	◯
avoiding removals	◯	◯	◯	◯	◯	◯
changing treatment strategy	◯	◯	◯	◯	◯	◯
reducing post-operative complications related to anatomy and position	◯	◯	◯	◯	◯	◯

-- PAGE BREAK --

Q28

Patient cases

The final part comprises four patient cases described with radiographs and related questions

Q29

Case 1.

Do you judge these two intraoral radiographs (IO) give adequate information (considering position of the mandibular canal, third molar root anatomy, and relation to the adjacent second molar) before a surgical removal of the mandibular third molar?

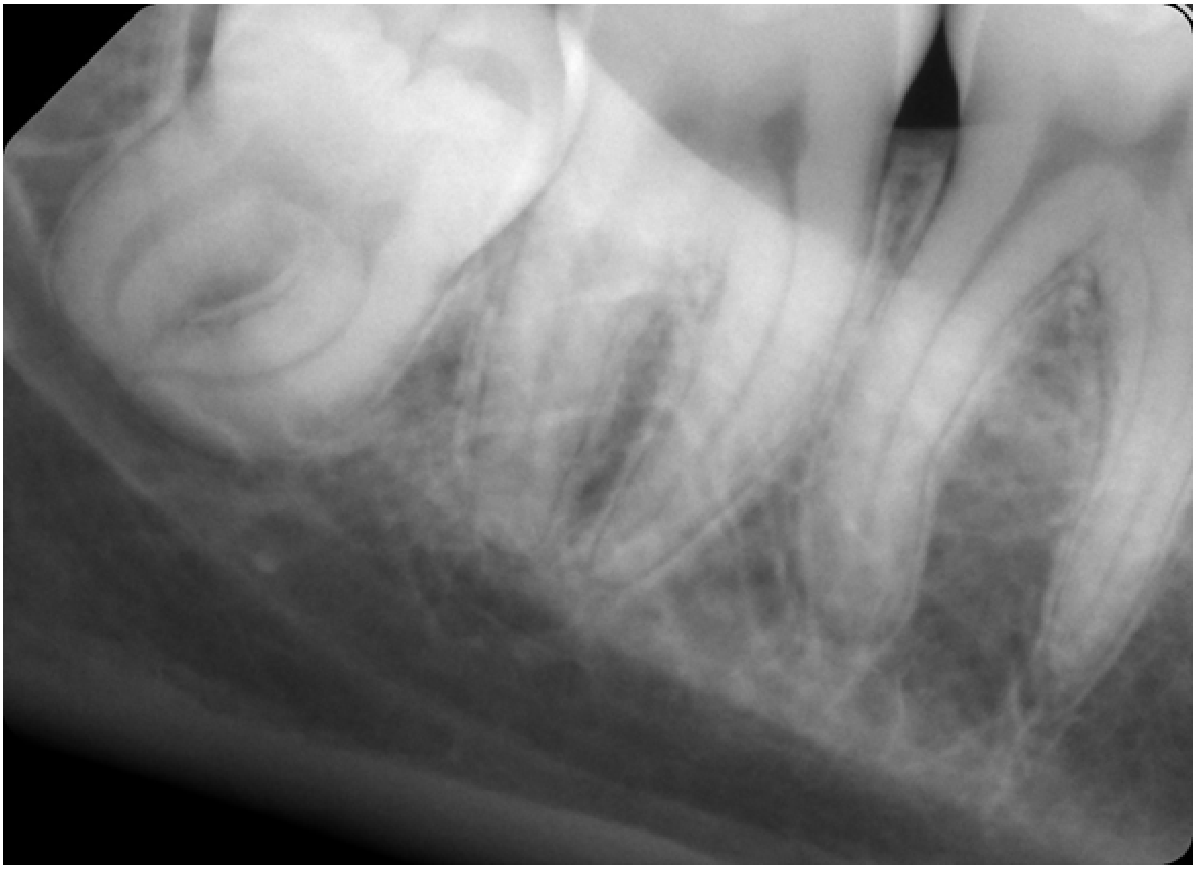

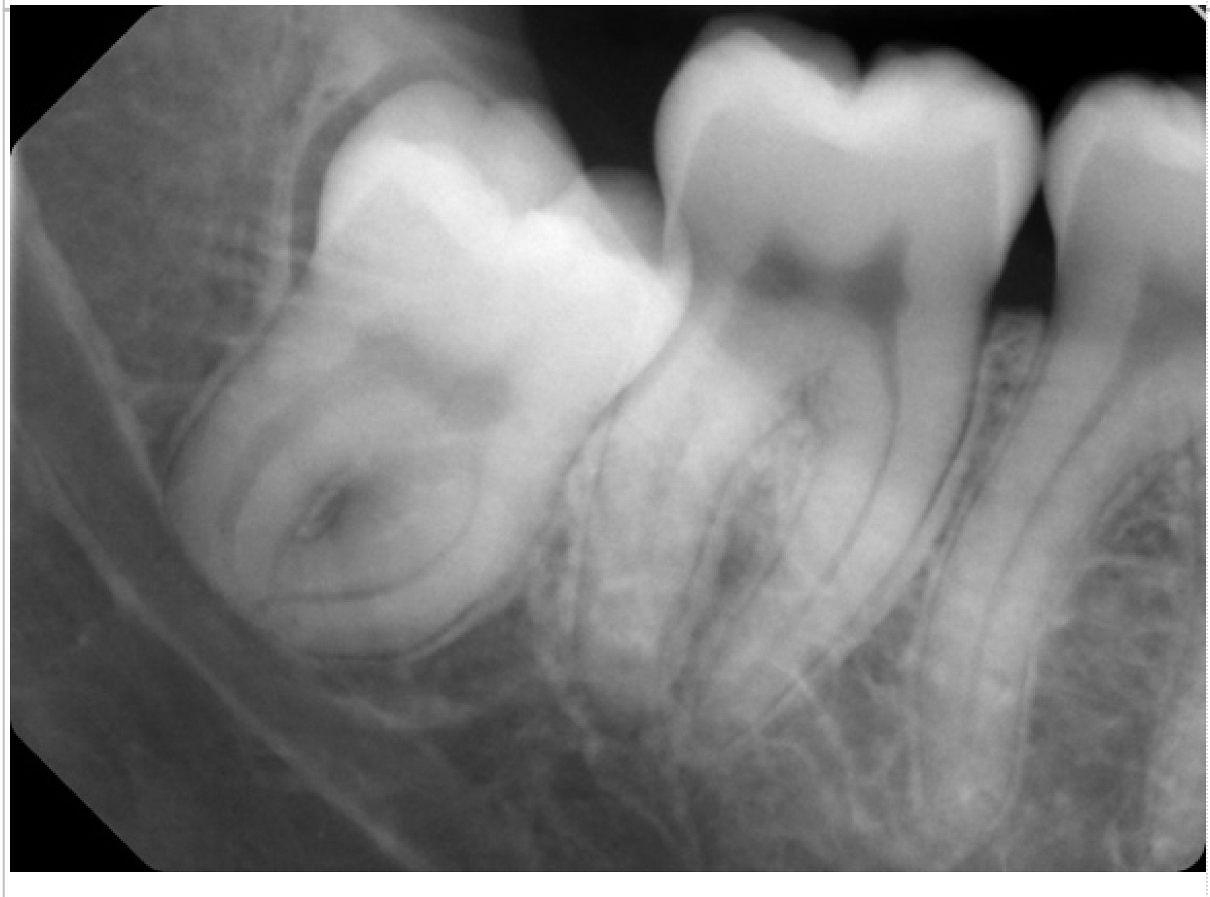

	Yes, IO suffices	No, first I wish to supplement with a panoramic radiograph	No, I wish to supplement with CBCT	Don't want to answer
Position of the mandibular canal	○	○	○	○
Root anatomy	○	○	○	○
Relation to the adjacent tooth	○	○	○	○

Q30

Rate your confidence to submit your answer

Not confident — Confident

Drag the slider to a point on the scale

-- PAGE BREAK --

Q31

Case 2.

Do you judge the panoramic radiograph (PAN) gives adequate information (considering position of the mandibular canal, third molar root anatomy, and relation to the adjacent second molar) before a surgical removal of the right mandibular third molar?

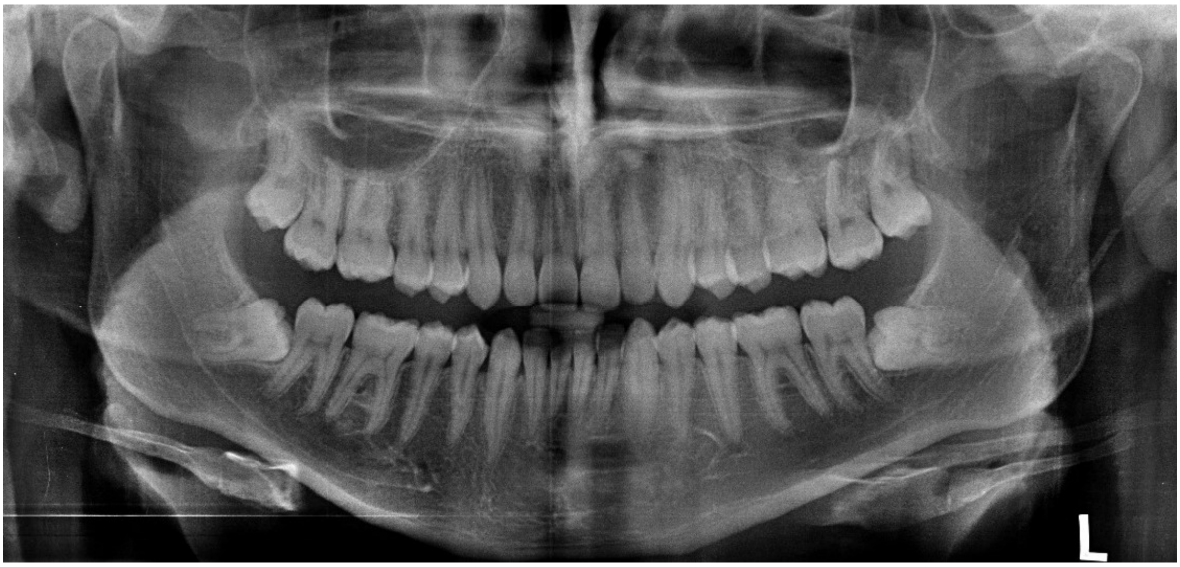

	Yes, PAN suffices	No, first I wish to supplement with intraoral radiographs	No, I wish to supplement with CBCT	Don't want to answer
Position of the mandibular canal	○	○	○	○
Root anatomy	○	○	○	○
Relation to the adjacent tooth	○	○	○	○

Q32

Rate your confidence to submit your answer

Not confident | Confident

Drag the slider to a point on the scale

-- PAGE BREAK --

Case 3:IO

If the answer to the subquestion Position of the mandibular canal **is exactly**

- No, first I wish to complete with intraoral radiographs

Or the answer to the subquestion Root anatomy **is exactly**

- No, first I wish to complete with intraoral radiographs

THEN PERFORM THE FOLLOWING ACTION

Show the following questions:

- Case 3. Continuing

Show the following questions:

- Rate your confidence to submit your answer

Case 3:CBCT

If the answer to the subquestion Position of the mandibular canal **is exactly**

- No, I wish to complete with CBCT

And the answer to the subquestion Root anatomy **is exactly**

- No, I wish to complete with CBCT

THEN PERFORM THE FOLLOWING ACTION

Show the following questions:

- Case 3. Continuing

Show the following questions:

- Rate your confidence to submit your answer

Q33

Case 3.

Do you judge the panoramic radiograph gives adequate information (considering position of the mandibular canal, third molar root anatomy, and relation to the adjacent second molar) before a surgical removal of the left mandibular third molar?

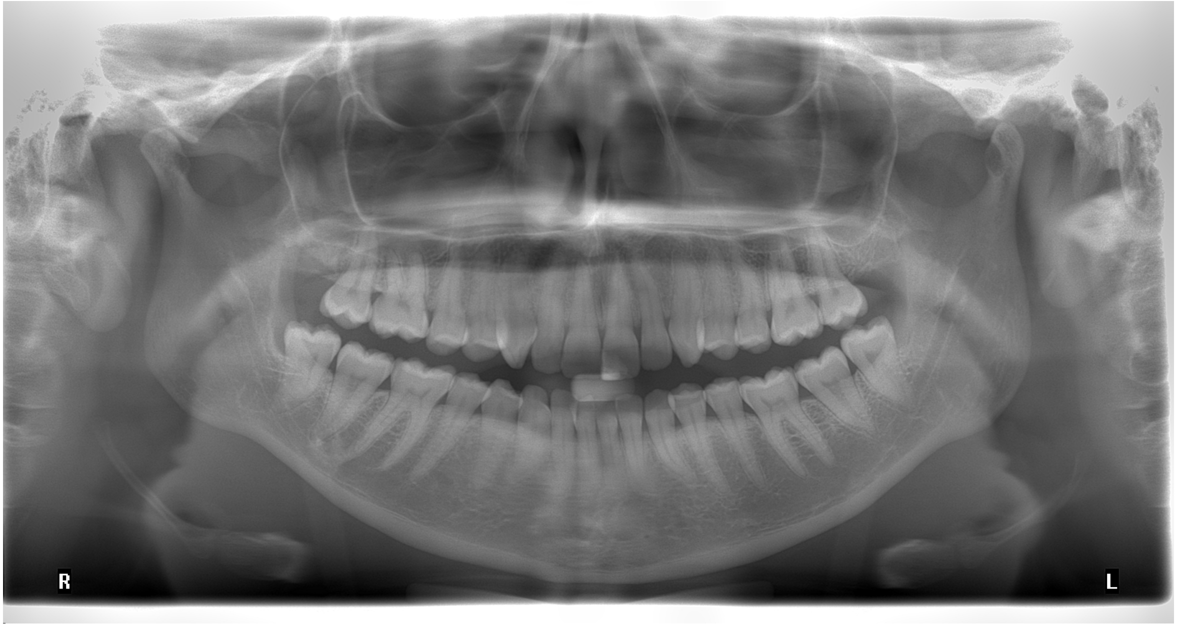

	Yes, PAN suffices	No, first I wish to supplement with intraoral radiographs	No, I wish to supplement with CBCT	Don't want to answer
Position of the mandibular canal	○	○	○	○
Root anatomy	○	○	○	○
Relation to the adjacent tooth	○	○	○	○

Q34

Rate your confidence to submit your answer

Not confident — Confident

Drag the slider to a point on the scale

-- PAGE BREAK --

Case 3:IO

If - Position of the mandibular canal **is exactly**

- No, first I wish to complete with intraoral radiographs

Or - Root anatomy **is exactly**

- No, first I wish to complete with intraoral radiographs

THEN PERFORM THE FOLLOWING ACTION

Show this question

Q35

Case 3. Continuing

Two supplemental intraoral radiographs are taken.

Do you judge these two intraoral radiographs together with the panoramic radiograph now give adequate information (considering position of the mandibular canal, third molar root anatomy, and relation to the adjacent second molar) before a surgical removal of the left mandibular third molar?

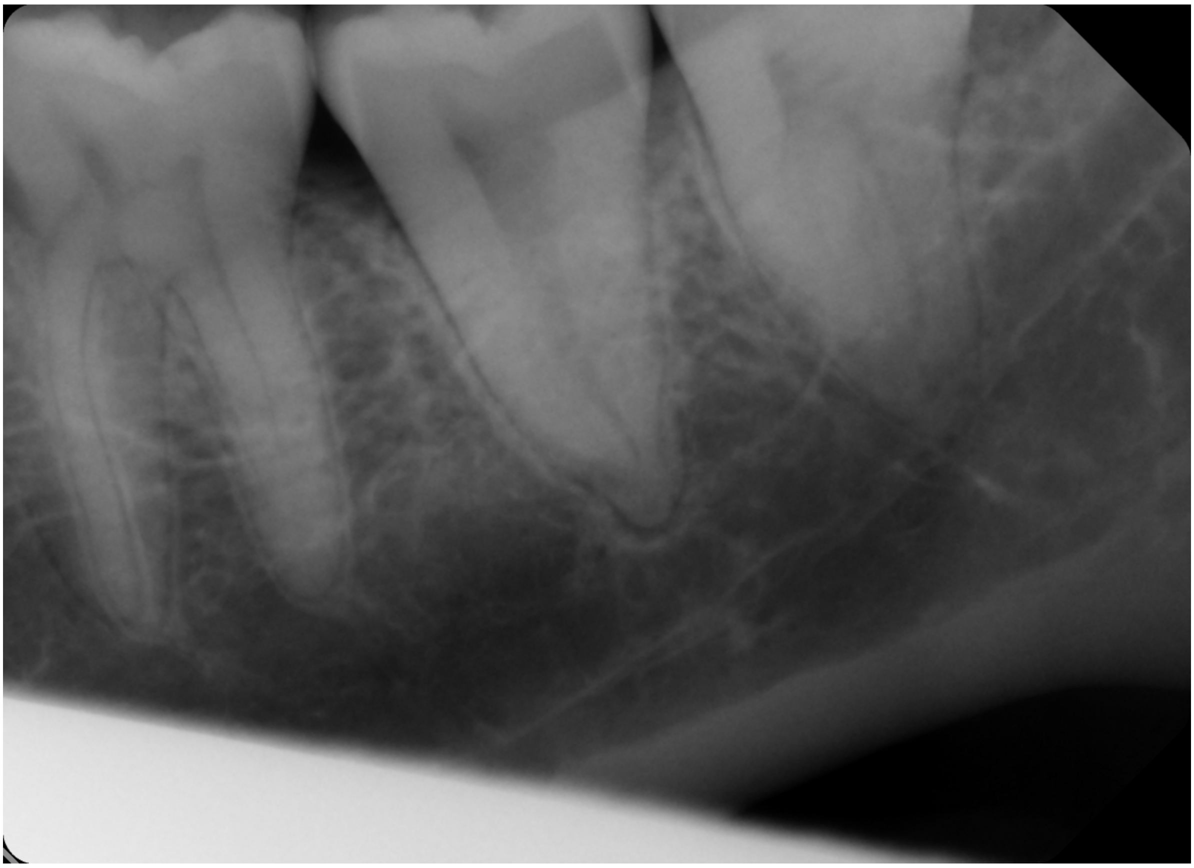

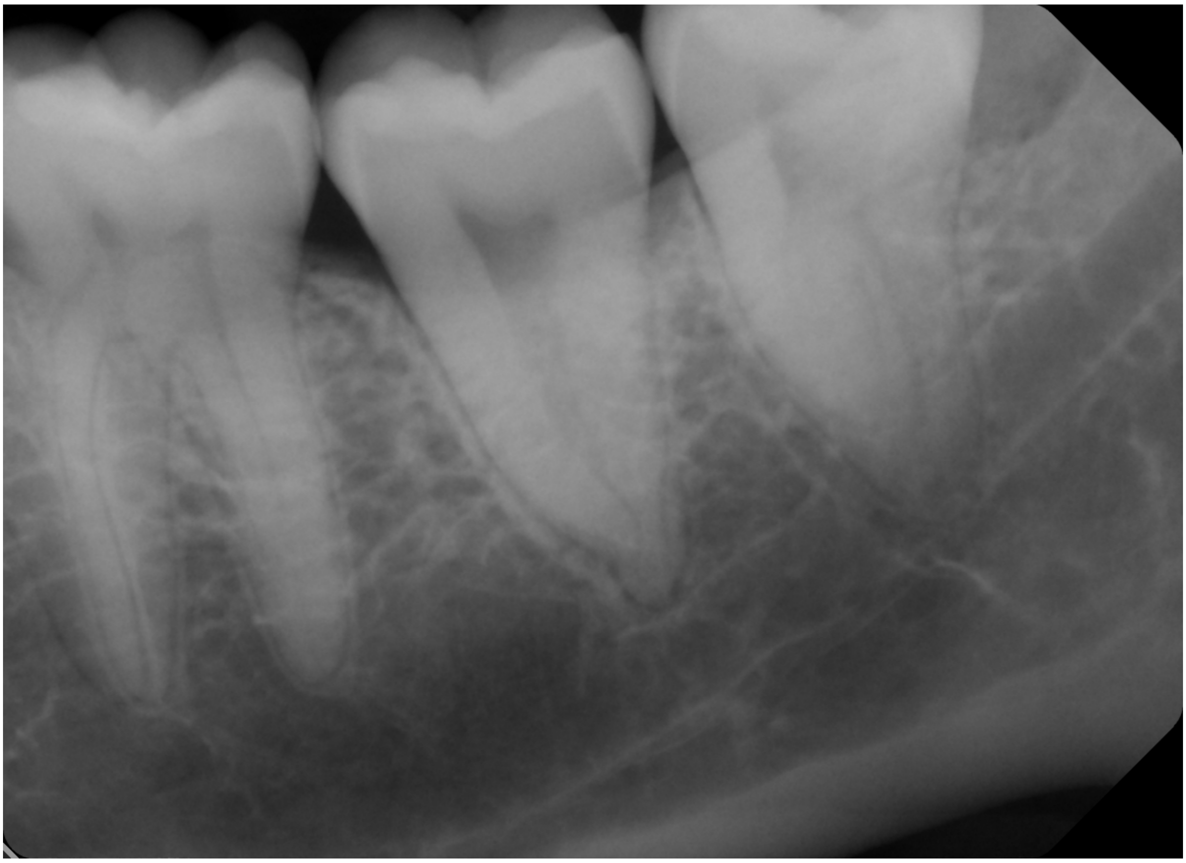

	Yes, IO and PAN suffice	No, I wish to supplement with CBCT	Don't want to answer
Position of the mandibular canal	◯	◯	◯
Root anatomy	◯	◯	◯
Relation to the adjacent tooth	◯	◯	◯

Case 3:IO

If - Position of the mandibular canal **is exactly**

- No, first I wish to complete with intraoral radiographs

Or - Root anatomy **is exactly**

- No, first I wish to complete with intraoral radiographs

THEN PERFORM THE FOLLOWING ACTION

Show this question

Q36

Rate your confidence to submit your answer

Not confident | Confident

Drag the slider to a point on the scale

Case 3:CBCT

If - Position of the mandibular canal **is exactly**

- No, I wish to complete with CBCT

And - Root anatomy **is exactly**

- No, I wish to complete with CBCT

THEN PERFORM THE FOLLOWING ACTION

Show this question

Q37

Case 3. Continuing

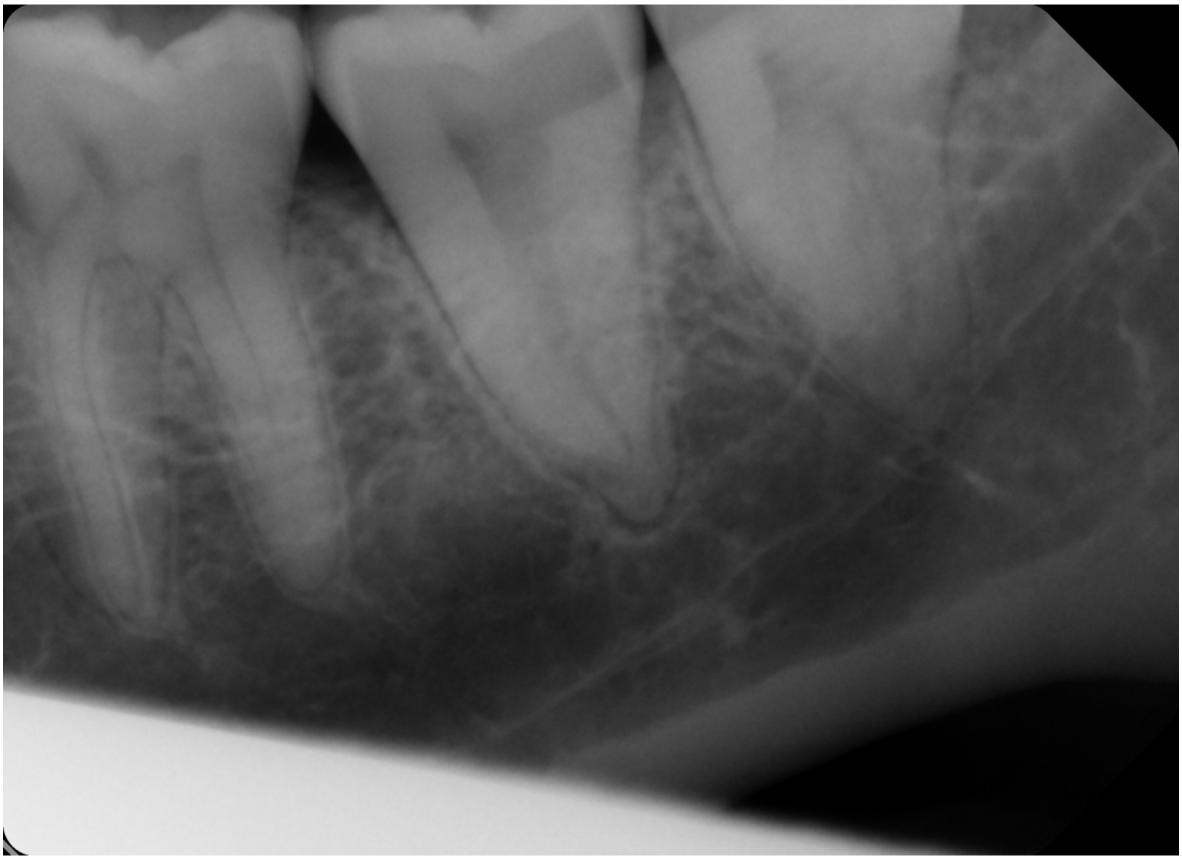

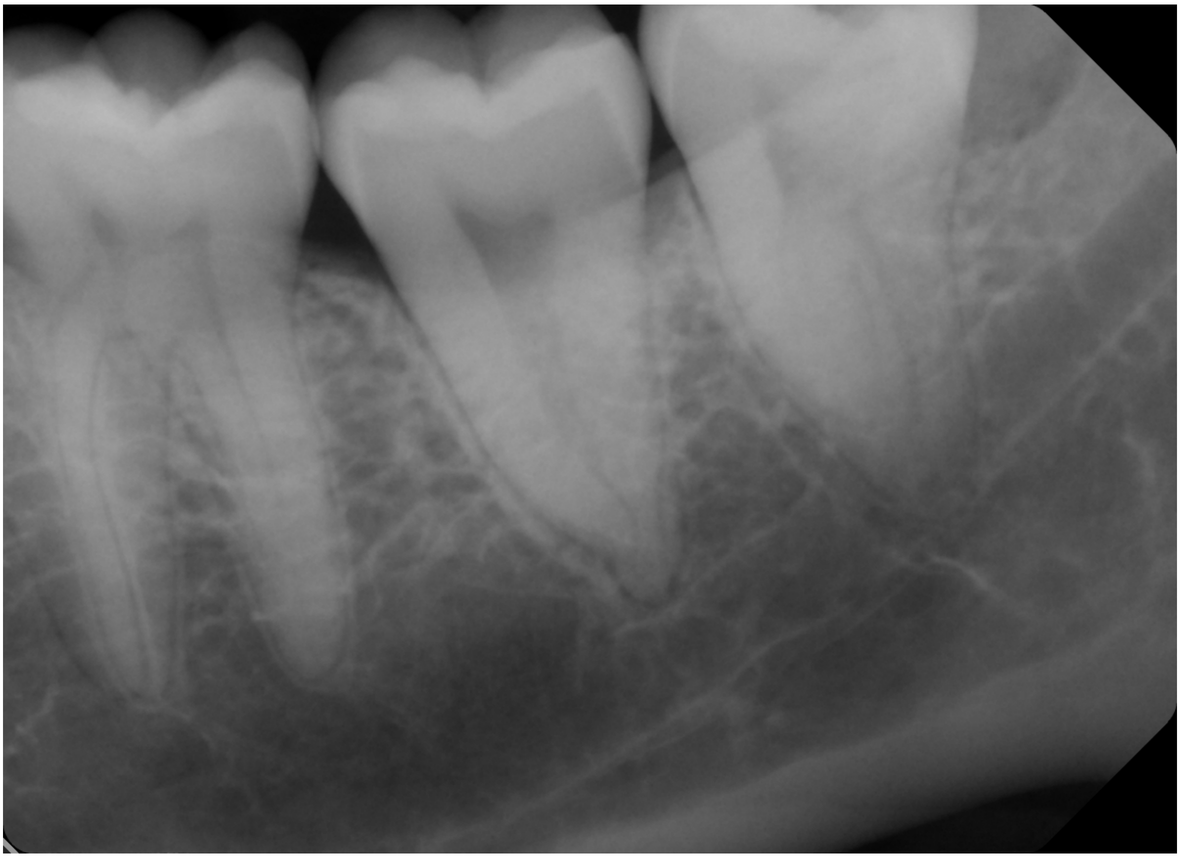

At further evaluation of already exposed radiographs you find these two intraoral radiographs of the right mandibular third molar.

Do you judge these two intraoral radiographs together with the panoramic radiograph now give adequate information (considering position of the mandibular canal, third molar root anatomy, and relation to the adjacent second molar) before a surgical removal of the left mandibular third molar?

	Yes, IO and PAN suffice	No, I wish to supplement with CBCT	Don't want to answer
Position of the mandibular canal	○	○	○
Root anatomy	○	○	○
Relation to the adjacent tooth	○	○	○

Case 3:CBCT

If - Position of the mandibular canal **is exactly**

- No, I wish to complete with CBCT

And - Root anatomy **is exactly**

- No, I wish to complete with CBCT

THEN PERFORM THE FOLLOWING ACTION

Show this question

Q38

Rate your confidence to submit your answer

Not confident | Confident

Drag the slider to a point on the scale

-- PAGE BREAK --

Q39

Case 4.

Do you judge the intraoral and panoramic radiographs together give adequate information (considering position of the mandibular canal, third molar root anatomy, and relation to the adjacent second molar) before a surgical removal of the right mandibular third molar?

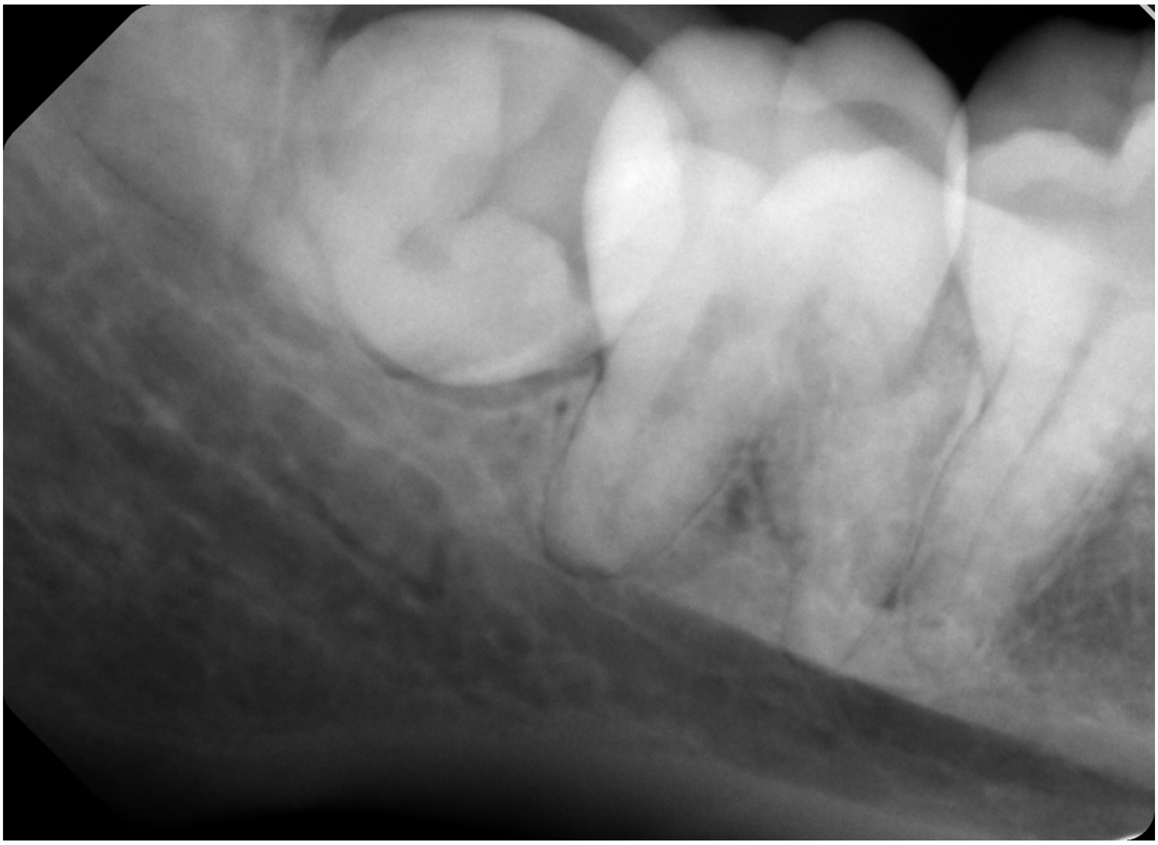

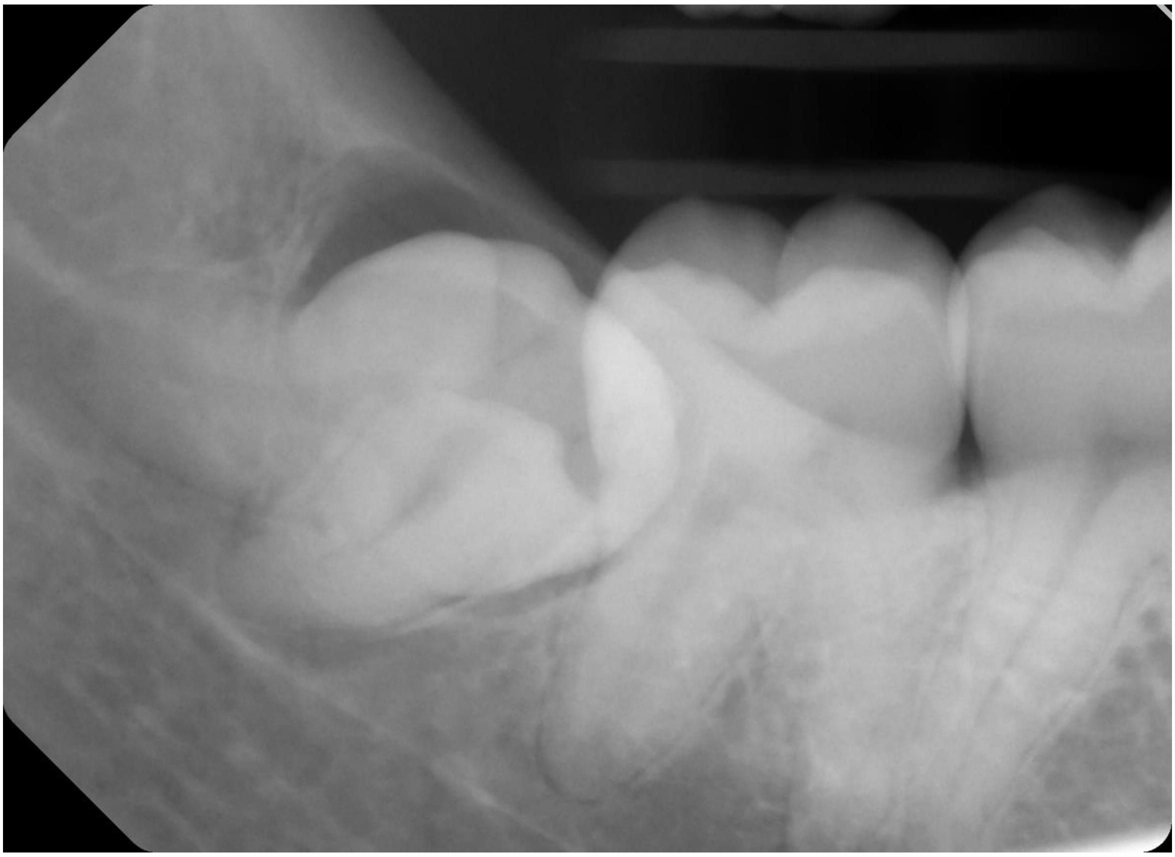

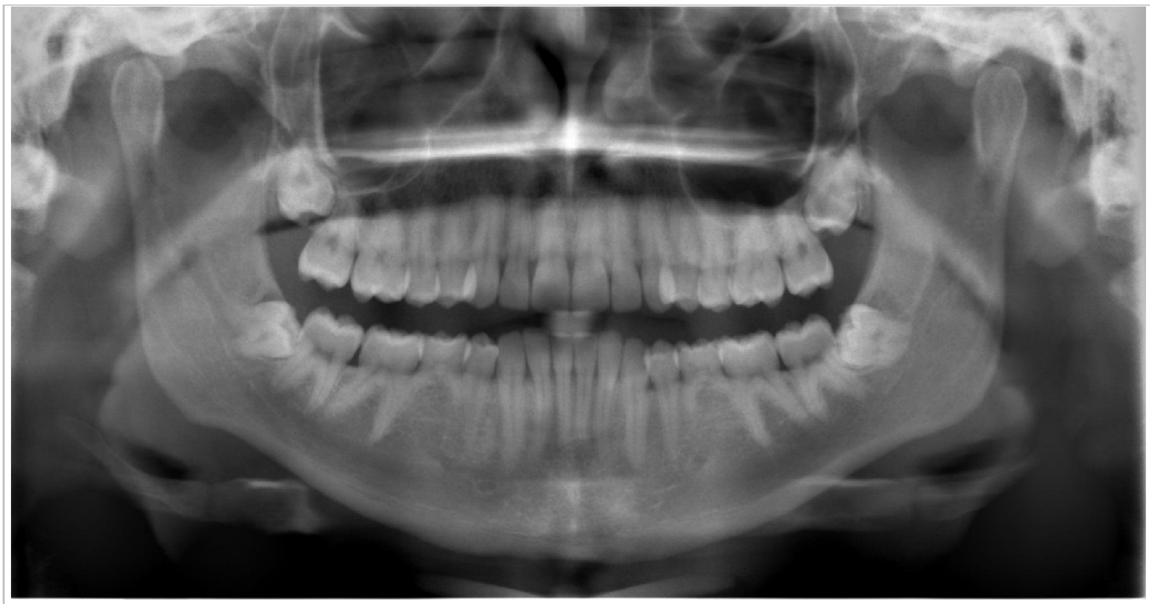

	Yes, IO and PAN suffice	No, I wish to supplement with CBCT	Don't want to answer
Position of the mandibular canal	○	○	○
Root anatomy	○	○	○
Relation to the adjacent tooth	○	○	○

Q40

Rate your confidence to submit your answer

Not confident — Confident

Drag the slider to a point on the scale

Q41

Thank you for taking your time and completing our survey.
Please submit your answers by clicking the "Send" button.

Your participation is important to us!
